# Common Genetic Variation Near the *Phospholamban* Gene Is Associated with Cardiac Repolarisation: Meta-Analysis of Three Genome-Wide Association Studies

**DOI:** 10.1371/journal.pone.0006138

**Published:** 2009-07-09

**Authors:** Ilja M. Nolte, Chris Wallace, Stephen J. Newhouse, Daryl Waggott, Jingyuan Fu, Nicole Soranzo, Rhian Gwilliam, Panos Deloukas, Irina Savelieva, Dongling Zheng, Chrysoula Dalageorgou, Martin Farrall, Nilesh J. Samani, John Connell, Morris Brown, Anna Dominiczak, Mark Lathrop, Eleftheria Zeggini, Louise V. Wain, for the The Wellcome Trust Case Control Consortium, The DCCT/EDIC Research Group, Christopher Newton-Cheh, Mark Eijgelsheim, Kenneth Rice, Paul I. W. de Bakker, for the QTGEN consortium, Arne Pfeufer, Serena Sanna, Dan E. Arking, for the QTSCD consortium, Folkert W. Asselbergs, Tim D. Spector, Nicholas D. Carter, Steve Jeffery, Martin Tobin, Mark Caulfield, Harold Snieder, Andrew D. Paterson, Patricia B. Munroe, Yalda Jamshidi

**Affiliations:** 1 Unit of Genetic Epidemiology and Bioinformatics, Department of Epidemiology, University Medical Center Groningen, University of Groningen, Groningen, the Netherlands; 2 Clinical Pharmacology and Barts and the London Genome Centre, William Harvey Research Institute, Barts and the London School of Medicine, Queen Mary University of London, London, United Kingdom; 3 Samuel Lunenfeld Research Institute, Mount Sinai Hospital, Toronto, Ontario, Canada; 4 Department of Genetics, University Medical Center Groningen, University of Groningen, Groningen, the Netherlands; 5 Wellcome Trust Sanger Institute, Wellcome Trust Genome Campus, Hinxton, Cambridge, United Kingdom; 6 Department of Twin Research and Genetic Epidemiology Unit, St Thomas' Campus, King's College London, St Thomas' Hospital, London, United Kingdom; 7 Cardiological Sciences, Division of Cardiac and Vascular Sciences, St George's University of London, London, United Kingdom; 8 Division of Clinical Developmental Sciences, St George's University of London, London, United Kingdom; 9 Department of Cardiovascular Medicine, University of Oxford, Wellcome Trust Centre for Human Genetics, Oxford, United Kingdom; 10 Department of Cardiovascular Sciences, University of Leicester, Glenfield Hospital, Leicester, United Kingdom; 11 BHF Glasgow Cardiovascular Research Centre, Division of Cardiovascular and Medical Sciences, University of Glasgow, Western Infirmary, Glasgow, United Kindom; 12 Clinical Pharmacology and the Cambridge Institute of Medical Research, University of Cambridge, Addenbrooke's Hospital, Cambridge, United Kingdom; 13 Centre National de Genotypage, Evry, France; 14 The Wellcome Trust Centre for Human Genetics, Oxford, United Kingdom; 15 Departments of Health Sciences & Genetics, University of Leicester, Leicester, United Kingdom; 16 Center for Human Genetic Research, Cardiovascular Research Center, Massachusetts General Hospital, Boston, Massachusetts, United States of America; 17 Program in Medical and Population Genetics, Broad Institute of Harvard and MIT, Cambridge, Massachusetts, United States of America; 18 NHLBI's Framingham Heart Study, Framingham, Massachusetts, United States of America; 19 Department of Epidemiology, Erasmus Medical Center, Rotterdam, The Netherlands; 20 Department of Biostatistics, University of Washington, Seattle, Washington, United States of America; 21 Division of Genetics, Department of Medicine, Brigham and Women's Hospital, Harvard Medical School-Partners HealthCare Center for Genetics and Genomics, Boston, Massachusetts, United States of America; 22 Institute of Human Genetics, Technical University Munich, Munich, Germany; 23 Institute of Human Genetics, Helmholtz Center Munich, Munich, Germany; 24 Istituto di Neurogenetica e Neurofarmacologia, CNR, Monserrato, Cagliari, Italy; 25 McKusick-Nathans Institute of Genetic Medicine, Johns Hopkins University, Baltimore, Maryland, United States of America; 26 Department of Cardiology, University Medical Center Groningen, University of Groningen, Groningen, the Netherlands; 27 Genetics and Genomic Biology, The Hospital for Sick Children, Toronto, Ontario, Canada; Queensland Institute of Medical Research, Australia

## Abstract

To identify loci affecting the electrocardiographic QT interval, a measure of cardiac repolarisation associated with risk of ventricular arrhythmias and sudden cardiac death, we conducted a meta-analysis of three genome-wide association studies (GWAS) including 3,558 subjects from the TwinsUK and BRIGHT cohorts in the UK and the DCCT/EDIC cohort from North America. Five loci were significantly associated with QT interval at P<1×10^−6^. To validate these findings we performed an *in silico* comparison with data from two QT consortia: QTSCD (n = 15,842) and QTGEN (n = 13,685). Analysis confirmed the association between common variants near *NOS1AP* (P = 1.4×10^−83^) and the phospholamban (*PLN*) gene (P = 1.9×10^−29^). The most associated SNP near *NOS1AP* (rs12143842) explains 0.82% variance; the SNP near *PLN* (rs11153730) explains 0.74% variance of QT interval duration. We found no evidence for interaction between these two SNPs (P = 0.99). PLN is a key regulator of cardiac diastolic function and is involved in regulating intracellular calcium cycling, it has only recently been identified as a susceptibility locus for QT interval. These data offer further mechanistic insights into genetic influence on the QT interval which may predispose to life threatening arrhythmias and sudden cardiac death.

## Introduction

The QT interval on the electrocardiogram (ECG) represents the period of ventricular depolarization and subsequent repolarisation. Individuals with delayed cardiac repolarisation show a longer QT interval and this predisposes them to the development of cardiac arrhythmias. Patients with the rare Mendelian Long QT Syndrome (LQTS) are at risk of sudden cardiac death [1]. Lengthening of the heart-rate corrected QT interval within the normal range is associated with increased coronary heart disease incidence and mortality, as well as all-cause mortality [2], [3]. QT prolongation is the most common cause for withdrawal or restriction of drugs that have already been marketed. Furthermore, many potentially valuable drugs fail to be approved or are downgraded to second-line status because they prolong QT and increase risk of serious life threatening arrhythmias, especially torsade de pointes [4].

QT interval length is known to be influenced by various parameters such as heart rate [5], age [6], sex [7], and medications [8], and studies have suggested that QT interval at the population level is a genetically influenced quantitative trait with up to 52% heritability [9]–[11]. Until recently, research into genetic factors influencing QT interval was limited to candidate genes known to have a role in arrhythmogenesis on the basis of their involvement in Mendelian Long or Short-QT Syndrome (LQTS or SQTS) [12]–[17]. However, an early genome-wide association (GWA) study [18] identified a common genetic variant (rs10494366) in the nitric oxide synthase 1 adaptor protein (*NOS1AP*) gene region, which has been consistently associated with QT-interval variation across many independent replication studies [19]–[24] The *NOS1AP* variant has been estimated to explain up to 1.5% of QT variance [18], therefore larger GWA studies of QT interval have the potential to detect additional common genetic variants, likely of more modest effect size. Recently, two consortia (QTGEN [25] and QTSCD [26]) reported meta-analyses of GWAS of QT interval duration in population-based cohorts, these papers describe a number of new loci [25], [26].

We report a meta-analysis of three GWA studies totalling 3,558 individuals and test for association between QT interval duration and approximately 2.4 million genotyped or imputed single nucleotide polymorphisms (SNPs). Subsequently, we performed an *in silico* comparison for our five most significant SNPs with QTGEN (n = 13,685) [25] and QTSCD (n = 15,842) [26]. Our results confirm the known association with the *NOS1AP* locus and QT interval duration, more importantly it confirms the recently reported association of variants near the *PLN* locus [25], [26]. We found no evidence of gene-gene interaction between *NOS1AP* and *PLN*.

## Results and Discussion

### Meta-analysis results from TwinsUK, BRIGHT and DCCT/EDIC cohorts

**Figure 1:**
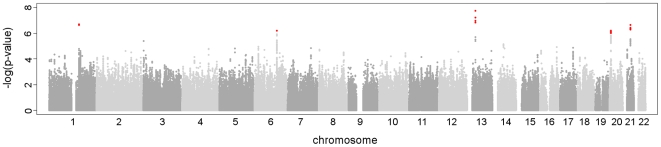
Manhattan plot for QT interval based on GWAS meta-analysis of TwinsUK, BRIGHT, and DCCT/EDIC cohorts. SNPs are ordered along the chromosomes on the x-axis. The −log10 (P) results are plotted for 2,399,142 SNPs of the meta-analysis of TwinsUK, BRIGHT and DCCT/EDIC cohorts for 3,558 individuals. The red dots indicate SNPs with P<10^−6^.

**Figure 2:**
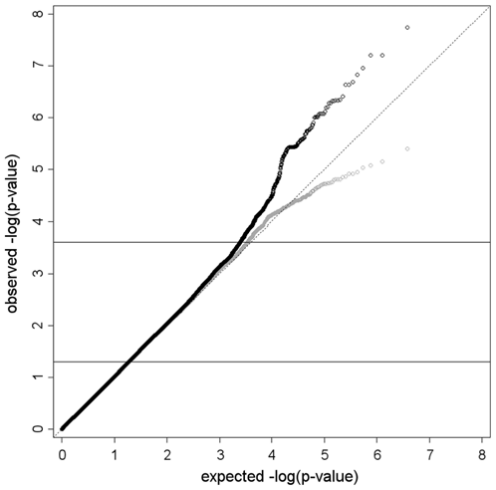
Quantile-quantile plots of association results of the meta-analysis from TwinsUK, BRIGHT and DCCT/EDIC cohorts. Based on 2,399,142 SNPs in 3,558 individuals from the combined cohorts. The −log10(P) plot of association test for QT interval is shown for all SNPs (black diamonds) and for all SNPs except those located within 1 Mb of the most significant SNPs of our five associated regions (dark grey) [26]. Genomic Control λ was 1.016 [25]. The lower horizontal line denotes the 95% percentile of the results of all SNPs, values lower than this threshold were used for calculating the λ. The upper line indicates the point from where P-values of the complete dataset deviate from the expected line.

The characteristics of the 3,558 individuals included in the meta-analysis are shown in Table 1. Genome wide genotyping was performed using a variety of platforms; therefore we imputed genotypes using the HapMap CEU sample. A total of 2,399,142 genotyped or imputed SNPs met the inclusion criteria for our study; we tested these for association with QT interval using an additive model. We observed highly associated SNPs in five chromosomal regions 1q23.3, 6q22.31, 13q13, 20p13 and 21q21.3 (Figure 1). Possible bias caused by population stratification was checked by calculating the genomic inflation factor λ of the meta-analysis [27], [28]. The λ was 1.016 indicating our samples showed little evidence for population stratification and therefore the results of the meta-analysis were not adjusted (Figure 2). Table 2 shows the results by cohort of the most significant SNP for each associated region, Table S1 shows the results for all SNPs with P<1×10^−6^. One SNP (rs885170) near *NBEA* on chromosome 13 exceeded the genome-wide significance threshold, P = 5×10^−8^ based on recent estimations of the genome-wide testing burden for common sequence variation [29], [30]. Four other SNPs had P<1×10^−6^. The first was rs12143842 (P = 2.1×10^−7^), it is located on chromosome 1, upstream of *NOS1AP*, a gene already identified as prolonging QT interval [18]. The second SNP rs2832357 (P = 2.3×10^−7^) is located on chromosome 21, near *GRIK1*, the third rs11153730 (P = 6.4×10^−7^) is located on chromosome 6 in an intergenic region in a cluster of SNPs near three genes *SLC35F1*, *C6orf204* and *PLN*. The final locus, rs6038729 (P = 6.3×10^−7^) is located on chromosome 20, near the *BMP2* gene.

**Table 1:** Study characteristics of the TwinsUK, BRIGHT and DCCT/EDIC cohorts.

	TwinsUK	BRIGHT	DCCT/EDIC
N	1,048	1,392	1,118
Age, mean (SD)	51.8 (12.0)	56.7 (10.9)	46.0 (7.0)
Sex, n (%) male	12 (1.1)	502 (36.0)	568 (51.0)
QT interval, mean (SD)	400.9 (27.9)	414.5 (33.8)	387.6 (29.2)
aHypertensive, %	21.8	100	50.8
bDiabetic, %	3.1	0	100

a Systolic blood pressure >140 mmHg or diastolic blood pressure >90 mmHg or taking anti-hypertensive drugs.

b Type 1 or type 2 diabetes.

**Table 2:** Results of the most significant SNP from the five regions associated with QT interval in GWAS meta-analysis of TwinsUK, BRIGHT and DCCT/EDIC cohorts.

SNP ID	Cohort	Coded Allele Frequency (%)	HWE (P)	Genotyped	Beta (SE)	R^2^ (%)^a^	P-value
rs12143842, chr 1	TwinsUK	24.4	0.11	No	0.22 (0.053)	1.76	3.2×10^−5^
coded allele: T	BRIGHT	26.6	0.89	No	0.15 (0.046)	0.83	0.0015
non-coded allele: C	DCCT/EDIC	25.3	0.68	Yes	0.085 (0.049)	0.27	0.082
	**Meta**	25.5	N/A	N/A	0.15 (0.028)	0.82	**2.1×10^−7^**
rs11153730, chr 6	TwinsUK	48.4	0.024	No	0.21 (0.145)	2.19	3.6×10^−6^
coded allele: C	BRIGHT	48.1	0.45	No	0.096 (0.04)	0.46	0.017
non-coded allele: T	DCCT/EDIC	49	0.75	No	0.075 (0.042)	0.28	0.076
	**Meta**	48.5	N/A	N/A	0.12 (0.024)	0.74	**6.4×10^−7^**
rs885170, chr13	TwinsUK	18.7	0.28	No	0.17 (0.058)	0.84	0.0045
coded allele: G	BRIGHT	19.7	0.41	No	0.22 (0.051)	1.58	1.2×10^−5^
non-coded allele: A	DCCT/EDIC	17.6	0.76	No	0.14 (0.057)	0.55	0.016
	**Meta**	18.8	N/A	N/A	0.18 (0.032)	0.99	**1.8×10^−8^**
rs6038729, chr20	TwinsUK	32.3	0.98	Yes	0.064 (0.046)	0.18	0.16
coded allele: C	BRIGHT	30.1	0.14	No	0.21 (0.044)	1.87	2.1×10^−6^
non-coded allele: A	DCCT/EDIC	32.3	0.78	Yes	0.11 (0.046)	0.54	0.015
	**Meta**	31.4	N/A	N/A	0.13 (0.026)	0.73	**6.3×10^−7^**
rs2832357, chr 21	TwinsUK	2.7	0.77	Yes	0.27 (0.16)	0.37	0.088
coded allele: G	BRIGHT	2.8	0.2	No	0.49 (0.12)	1.29	5.3×10^−5^
non-coded allele: A	DCCT/EDIC	2.6	0.21	Yes	0.38 (0.13)	0.71	0.0031
	**Meta**	2.7	N/A	N/A	0.40 (0.076)	0.82	**2.3×10^−7^**

HWE: Hardy-Weinberg equilibrium test; SE: standard error; N/A: not applicable.

a Percentage of explained variance.

### Results for known LQTS and SQTS candidate genes

There are 11 genes identified to date as being causative for Mendelian single gene forms of LQTS and SQTS. Notably, both of the recent GWAS meta-analyses [25], [26] found that common variants in a subset of these genes encoding ion channels, known to cause the Mendelian LQTS, were the most strongly associated with QT interval. We looked up the SNP with the lowest P-value in each of these genes and up to 20 kb upstream and downstream. Only one SNP in *KCNE1* (LQT5; rs3787730 A>G; frequency allele A: 31.7%; β≈−1.6 ms/allele A; P = 0.00045; Table S2) was found to be significantly associated with QT interval, although not genome-wide significant. This SNP was not in linkage disequilibrium with the polymorphisms D85N (rs1805128; r^2^ = 0.011; P = 0.87) and rs727957 (r^2^ = 0.010; P = 0.090), which were previously found to be associated with prolonged QT interval in a general population [14], [31]. None of the other genes showed evidence for association with QT interval in our study.

### Follow-up of the top 5 loci

To validate potential associations with QT interval we selected the most associated SNP in each of the five regions from the primary meta-analysis and conducted an *in silico* comparison with data from the QTSCD and QTGEN consortia (Table 3). This confirmed two of our five loci as being significantly associated with QT interval in the replication at P = 5×10^−8^; the strongest evidence of association was with a SNP near *NOS1AP*, rs12143842 (P = 1.4×10^−83^). rs10494366 is the *NOS1AP* polymorphism most commonly associated with QT interval in previous studies [19]–[24], it reached a P-value of 0.035 in our data-set. This SNP is not strongly correlated to rs12143842 in the HapMap CEU samples (r^2^ = 0.102). The rs12143842 polymorphism explains 0.82% variance of QT interval in our meta-analysis.

**Table 3:** Results of the five most significant loci from GWAS meta-analysis of TwinsUK, BRIGHT and DCCT/EDIC cohorts and *in silico* comparison with the QTGEN and QTSCD consortia data.

SNP	Chr	Position^a^	Flanking genes (distance to SNP in kb)	Coded Allele	Meta-analysis of TwinsUK, BRIGHT and DCCT/EDIC	QTSCD	QTGEN	META
rs12143842	1	160,300,514	*OLFML2B*; *NOS1AP*	Freq T (%)	26	24	26	25
			(−40.2; 5.7)	Beta	0.15	0.16	0.21	0.18
				P-value	2.1 10^−7^	1.6 10^−35^	8.1 10^−46^	**1.4 10^−83^**
rs11153730	6	118,774,215	*SLC35F1*;*C6orf204;PLN*	Freq C (%)	48	50	50	50
			(−28.7; intronic; 202.0)	Beta	0.12	0.091	0.08	0.09
				P-value	6.4 10^−7^	5.2 10^−16^	5.3 10^−10^	**1.9 10^−29^**
rs885170	13	34,095,789	*RFC3*; *NBEA*	Freq G (%)	19	20	20	20
			(−657.1; 318.7)	Beta	0.18	−0.011	−0.0051	0.01
				P-value	1.8 10^−8^	0.44	0.76	**0.28**
rs6038729	20	7,085,757	*BMP2*; *FUSIP1P2*	Freq C (%)	31	32	31	32
			(−367.8; 675.3)	Beta	0.13	0.020	−0.0042	0.023
				P-value	6.3 10^−7^	0.085	0.76	**0.0071**
rs2832357	21	29,785,765	*BACH1*; *GRIK1*	Freq G (%)	3	3	3	3
			(−129.7; 45.4)	Beta	0.40	0.0075	0.022	0.053
				P-value	2.3 10^−7^	0.82	0.58	**0.031**

Freq: allele frequency.

a NCBI Genome build 36.3.

**Figure 3:**
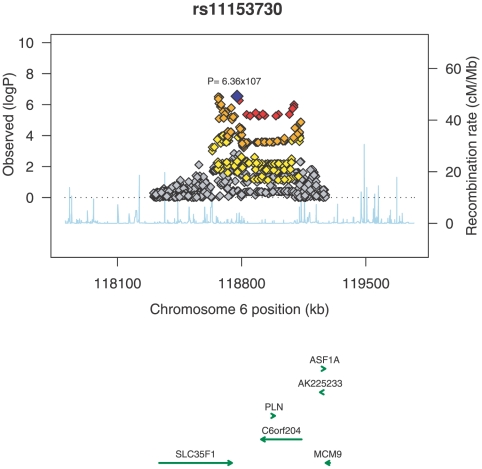
Regional association plot for the *SLC35F1/C6orf204/PLN* locus on chromosome 6. Shown is the region extending to 500 kb either side of the most associated SNP rs11153730. The SNPs are illustrated on −log10(*P*) scale as a function of chromosomal position (NCBI build36.3). The sentinel SNP is illustrated in blue. Surrounding SNPs are coloured according to their r^2^ with rs11153730 (red indicates an r^2^>0.8, orange an r^2^ of 0.5–0.8, yellow an r^2^ of 0.2–0.5 and grey an r^2^ of less than 0.2).

The second significantly associated locus was at chromosome 6q22.31, near the *SLC35F1/C6orf204/PLN* loci (rs11153730; P = 1.9×10^−29^, Table 3; Figure 3). This SNP is intergenic in a region with only a few genes. Little is known about *SLC35F1* and *C6orf204*, however the most plausible candidate gene is phospholamban (*PLN*), an inhibitor of the Ca^2+^-ATPase isoform 2a (SERCA2a), a Ca^2+^ transporting intracellular pump located in the sarcoplasmic reticulum (SR) of cardiac muscle cells. The most associated SNP rs11153730 is strongly correlated with two intronic SNPs (rs3752581 and rs13192336) in *PLN* (r^2^ of 0.7 in HapMap CEU samples). Both SNPs are associated with QT interval P = 7.3×10^−4^; both imputed.

The frequency of the C allele of rs11153730 near *PLN* was consistent across studies (48.4% in TwinsUK; 48.1% in BRIGHT; 49.0% in DCCT/EDIC). Each C allele prolongs the standardized QT interval by 0.122 units (corresponding to ∼2.5 ms) and explains 0.74% variance of QT interval duration (Table 2). The effect size from combining all studies was lower, 0.09 with 0.40% explained variance (Table 3). This decrease in effect size is not unexpected and may be attributed to the “winner's curse” phenomenon [32].

The effects of the *NOS1AP* and *PLN* loci did not show significant heterogeneity between the three studies as tested by the Q test (P>0.05, Table S1) [33], in total the two most significant loci in our initial meta-analysis explain c. 1.6% of the variance in QT interval duration. We also investigated whether there was any evidence for a gene-gene interaction between the two most significantly associated SNPs in the *NOS1AP* (rs12143842) and *PLN* (rs11153730) genes. Analysis revealed no evidence to suggest this (P = 0.99).

### Phospholamban and QT interval length

Phospholamban (in its unphosphorylated state) is an inhibitor of the Ca^2+^-ATPase isoform 2a (SERCA2a), a Ca^2+^ transporting intracellular pump located in the SR of cardiac muscle cells. The SR controls contraction and relaxation by regulating intracellular calcium levels. Phosphorylation of PLN reduces inhibition of SERCA2a, leading to activation of the Ca^2+^ pump, enhanced muscle relaxation rates and decreased Ca^2+^ levels, thereby contributing to the contractile response elicited by beta-agonists [34], [35].

*PLN* knock-out mice exhibit increased rates of basal myocardial contraction as well as increased rates of basal myocardial relaxation [34], [35]. However, the enhanced contractility observed with *PLN* knockout mice is in contrast to humans lacking PLN who develop a lethal cardiomyopathy. Indeed, several rare (non-HapMap) mutations in the human *PLN* gene have been associated with either dilated [36] or hypertrophic cardiomyopathy [37], presumably caused by PLN mediated over-inhibition [38], [39] or chronic activation of SERCA2a [37] respectively. Interestingly, some of the individuals in the study of Haghighi *et al*. [40] who were heterozygous for an Arg14Del mutation presented with ventricular extra systolic beats and ventricular tachycardia.

It has previously been shown that prolongation of cardiac repolarization elevates intracellular Ca^2+^, potentially increasing the risk of arrhythmias [41]. Del Monte *et al*. [42] reported that over-expression of SERCA2a in rats reduced ventricular arrhythmias in an ischemia/reperfusion model. Recent evidence showed that intracellular Ca^2+^ may also influence K^+^ currents and, thus duration of the action potential [43]. Suppression of SERCA2a by PLN may reduce SR Ca^2+^ content and lead to QT interval shortening through calmodulin kinase II-dependent alterations in K^+^ currents [44], whereas SERCA2a over-expression may result in an increased Ca^2+^ content and QT interval prolongation as shown in mice without underlying cardiac disease [43].

In addition to PLN, neuronal nitric oxide synthase (NOS1) is also involved in regulating intracellular calcium cycling [45]. NOS1AP is a regulator of NOS1. Furthermore, a recent study of transgenic mice with cardiomyocyte-specific *NOS1* over-expression suggested that the greater intracellular Ca^2+^ transients, and SR Ca^2+^ load in these mice following treatment to induce cardiac hypertrophy could be explained, at least in part, by modulation of PLN phosphorylation status [46]. In fact, nNOS-derived NO has been shown to regulate myocardial relaxation and intracellular Ca^2+^ decay by promoting PKA-mediated PLN phosphorylation [47] and in nNOS^−/−^ myocytes, decreased PLN phosphorylation has been shown to decrease the rate of SR Ca^2+^ reuptake and impair relaxation by inhibiting SERCA2a activity. Whether abovementioned mechanisms are similar in humans awaits confirmation in future studies.

In addition to interaction with the SERCA genes (*ATPT2A1* and *ATP2A2*) and NOS1, it has also been suggested that PLN interacts at the protein level with a number of molecules involved in ATP-dependent transport of Ca^2+^ (Figure S1). Furthermore, PLN is highly expressed in muscle and heart tissue and is co-expressed with muscle, or heart specific genes (Figures S2 and S3). Together with the data described above these observations suggest that PLN is most likely to influence QT interval through regulation of myocellular calcium cycling.

The current findings indicate that maintaining normal homeostatic calcium cycling is crucial as when imbalanced it can lead to human heart failure. However, *PLN* may also play a role in cardiac repolarization, which again if disturbed leads to serious arrhythmias. Earlier studies have suggested screening for *PLN* mutations in individuals with dilated cardiomyopathy [48], [49]. In view of the fact that both super-inhibition, as well as, over-expression of SERCA2a by PLN may lead to cardiomyopathy and heart failure, it maybe that any therapies directed at PLN will be challenging to develop without disturbing the fine balance between SERCA2a and PLN.

Apart from the discovery of variants in genes causing LQTS and SQTS [13], [14], [25], [26], [50] and *NOS1AP* [18]–[26], very few variants have thus far been consistently associated with QT interval duration in the general population. Our study with 3,558 individuals illustrates the potential of GWAS to identify novel variants playing a role in determining QT interval duration. Our results highlight the consistent role of *NOS1AP* genetic variants in modulating QT interval and confirm the recently identified *PLN* locus. Despite only two loci reaching genome-wide significance overall, and their effects, although positive are modest (<1% of variance), these results must be considered in the context of our sample size. Meta-analyses of larger datasets will no doubt identify additional SNPs with smaller effects or with rarer allele frequencies associated with QT interval.

In summary, our study is amongst the first to report common variants near *PLN* associated with QT interval. Functional relevance of PLN to QT interval duration is supported, it has a well documented role in myocellular calcium cycling, our results suggest that further molecular and functional analyses of this gene is warranted to pursue its role in regulating QT interval duration. Furthermore, genetic variation in the *NOS1AP* gene has also been associated with risk of mortality in patients using both cardiac and non-cardiac drugs [51], [52]. Therefore the observed association between *PLN* and QT interval may also have implications for cardiac and non-cardiac drug development, as QT prolongation is a very common reason for cessation of development or withdrawal of drugs. Further investigation into the potential interaction between *PLN* variants and drug-induced QT prolongation would also be of great interest.

## Materials and Methods

### Ethics Statement

All subjects involved in the study gave fully informed written consent for the collection of samples and subsequent analysis. The TwinsUK study received written ethical approval for this study from the National Research Ethics Service (St. Thomas' Research Ethics Committee Ref. EC04/015). The BRIGHT study received written ethical approval from The London Multicentre Research Ethics Committee. The DCCT/EDIC study received written ethical approval from The Hospital for Sick Children Research Ethics Board.

### Study subjects and SNP genotyping

#### The TwinsUK Study

Samples from the TwinsUK cohort were genotyped with the Infinium assay (Illumina, San Diego, USA) across three fully compatible SNP arrays, the Hap300 Duo, Hap300, and Hap550 [53]. SNP calling was performed using the Illuminus software [54]. SNPs were excluded if they violated Hardy–Weinberg equilibrium (HWE) (p<1.0×10^−4^); had genotype call rates <90%; or had a minor allele frequency (MAF) of less than 0.01. Individuals were excluded if the sample call rate was less than 95%, autosomal heterozygosity was not between 33 and 37%, genotype concordance was over 97% with another sample and the sample was of lesser call rate, non-caucasian ancestry either self-identified or identified by cluster analysis in STRUCTURE [55], or unexplained relatedness (estimated proportion of allele shared identical by descent >0.05 [56]) to >120 other samples. This resulted in GWAS data being available on 305,912 SNPs for 2,256 individuals from 595 dizygotic (DZ) twin pairs and 1066 singletons (among them twins from monozygotic (MZ) twin pairs) from the TwinsUK cohort. This cohort was previously shown to be representative of the general (singleton) UK population [57]. ECG data were available on 1,104 of these individuals. Eight hundred and sixty had automated measurements of the QT interval by the Cardiofax ECG-9020K (Nihon Kohden UK Ltd., Middlesex, UK) and 244 were scored manually using a high-resolution digitizing board (GTCO CalComp Peripherals, USA).

Fifty six individuals were removed from the data set because of atrial fibrillation, QRS duration >120 ms or presence of a heart condition (i.e. ischemic heart disease, stroke or bypass surgery). None of the genotyped twins had a pacemaker or used anti-arrhythmic drugs. The dataset for analyses consequently included 1,048 individuals, of which 588 were DZ twins (i.e. 294 pairs) and 460 singletons. These singletons included 235 MZ twins of which the mean QT interval of both twins was used to optimise information.

#### The BRIGHT study

Two thousand unrelated white European hypertensive individuals from the BRIGHT study (www.brightstudy.ac.uk) were genotyped with the GeneChip Human Mapping 500K Array Set (Affymetrix). Only individuals and SNPs passing WTCCC thresholds for quality control [58] were included in the analysis. Briefly, individuals were excluded if they had >3% missing data or evidence of non-Caucasian ancestry under Eigenstrat analysis [59]. SNPs were excluded if they showed deviation from HWE (p<5×10^−7^), high levels of missing data (capture rate <95%) or low MAF (<1%). Twelve-lead ECG recordings (Siemens-Sicard 440; http://www.brightstudy.ac.uk/info/sop04.html), which produces an automated measurement of the QT interval, were available for all subjects. All data were transferred from each recruitment centre by electronic modem to electrophysiologists from the West of Scotland Primary Prevention Study (Professor Peter MacFarlane) for central reporting. Thirteen hundred and ninety two individuals remained in the analysis after exclusion of those having ischemic disease, stroke, or bypass, atrial fibrillation, or QRS duration >120 ms and having full covariate information.

#### The DCCT/EDIC Study

The Diabetes Control and Complicatons Trial (DCCT)/Epidemiology of Diabetes Interventions and Complications (EDIC) study was a clinical trial and follow-up of subjects with type 1 diabetes. Fourteen hundred forty one patients with type 1 diabetes were recruited for the DCCT [60] and followed-up in EDIC [61]. Genome-wide genotyping in subjects from the DCCT/EDIC was performed using the Illumina 1 M beadchip assay (Illumina, San Diego, USA) of which 841,342 SNPs with a MAF>1% were subsequently analyzed statistically. Autosomal SNPs showing significant association with gender (p<10^−8^) or deviating from HWE (p<10^−8^) were excluded from the analysis. To reduce the possibility of population stratification, we limited the analysis to individuals who self-identified as white, and excluded individuals who were determined to be admixed between Caucasian and other ethnic groups through population genetic approaches, using Eigenstrat [59] seeding with genotype data from the three major populations genotyped in HapMap Phase II [62].

Twelve-lead resting ECGs were obtained by a certified technician or research nurse at 29 clinics, measured digitally and read according to the revised Minnesota Code at the Central ECG Reading Unit (University of Minnesota, under the direction of Dr. Richard S. Crow) [63]. In brief, at least 1 full min of ECG tracing was obtained consisting of 5 s of each of the leads (I, II, III, aVR,aVL, aVF, and V1–V6). Additionally, individuals having ischemic disease, stroke, or bypass, atrial fibrillation, or QRS duration >120 ms were excluded, therefore in total 323 individuals were excluded from the analysis.

### Imputation

As all three cohorts used different platforms for genome wide genotyping, non-genotyped autosomal SNPs were imputed. For TwinsUK and BRIGHT individuals imputation was performed using Phase II CEU HapMap data (release 22, build 36) as the reference database using IMPUTE version 0.3.2 [64]. For DCCT/EDIC individuals imputation was performed using Phase II CEU HapMap data (release 22, build 36) using MACH v 1.0.16 [62], [65].

### Statistical Analyses

Using regression analysis, we adjusted QT interval for RR interval, age, sex, height, body mass index, hypertension, and QT interval shortening or prolonging drugs (if available) within each cohort. For the TwinsUK sample, an extra covariate for the method of measurement (automatically vs. manually scored) of the QT interval was incorporated. Standardized QT interval residuals were used for further analyses. For the TwinsUK and BRIGHT cohorts, association between standardized corrected QT interval data and autosomal SNPs was tested with an F-test in SNPTEST version 1.1.4 using an additive model and the proper option to account for the uncertainty of the genotypes that were imputed [64]. As the TwinsUK cohort data consisted partly of dizygotic twins, the variances of the regression coefficients were corrected for the sibship relations using the Huber-White method for robust variance estimation in R [66], [67]. In the DCCT/EDIC cohort, SNPs were tested for association with corrected QT interval using an additive model in MACH2QTL version 1.04 [62], [65]. Genomic control was performed to check for population stratification [27].

A meta-analysis was conducted in R using the inverse variance-weighted fixed effects method on the beta estimates relative to a consistent reference allele to combine the results of TwinsUK, BRIGHT, and DCCT/EDIC cohorts (own software). Only SNPs with MAF>1%, P>10^−6^ for the HWE test calculated using the genotypes inferred after imputation by maximum likelihood expectation and an imputation quality score reflecting the observed by expected variance ratio >0.5 for TwinsUK and BRIGHT (IMPUTE proper_info) and >0.3 for DCCT/EDIC (MACH r^2^) were included in the analysis. Heterogeneity of observed effects was tested by the Q test [33].

### QTSCD and QTGEN ‘in silico’ cohorts: description, genotyping and analysis

The QTSCD consortium conducted a meta-analysis of results of GWAS on QT interval from the ARIC, SardiNIA, KORA, GenNOVA and HNR cohorts comprising in total 15,842 individuals (in press [26]). The QTGEN consortium combined the results of GWAS on QT interval from the Framingham Heart Study, the Rotterdam Study, and the Cardiovascular Health study in a meta-analysis including in total 13,685 individuals (in press [25]). Both studies imputed genotype data in order to facilitate the comparison of genotyping results across different platforms. Further details of materials and methods of both consortia can be found elsewhere (in press [25], [26]).

We performed a meta-analysis combining our results with those of the QTSCD and the QTGEN consortia for our five most statistically significant independent SNPs using methods as described above.

## Supporting Information

Figure S1 PLN protein-protein interaction network. This protein-protein interaction network was generated by the STRING program (http://string.embl.de) after querying the PLN gene using a high confidence score (0.700). Data supporting the interactions illustrated were derived from experimental studies (purple lines), databases (blue lines) and text mining (green lines). The genes that are involved in the calcium signaling pathway are indicated in red, the nodes with purple stars indicate the genes that are associated with cardiovascular diseases as based on functional annotation by DAVID (http://david.abcc.ncifcrf.gov). Only for large nodes are 3D protein structures available in STRING. The colour of the nodes does not encode any information.
(0.22 MB DOC)

Figure S2 PLN co-expression network. This network is retrieved from the gene co-expression database COXPRESdb (http://coxpresdb.hgc.jp) using the PLN Entrez ID (5350) as the query. The co-expression network is drawn based on rank of correlation from 123 Human microarray experiments released by the NCBI GEO database. The bold grey lines indicate average ranks from 1 to 4. The normal light gray lines indicate average ranks from 5 to 29. The orange lines indicate conserved co-expression based on evidence from the NCBI HomoloGene database and COXPRESdb. The gene names in red indicate muscle or heart specific expression and nodes with purple stars refer to genes that are associated with cardiovascular diseases as based on functional annotation by DAVID (http://david.abcc.ncifcrf.gov). For large nodes 3D protein structures are available in STRING. The colour of the nodes does not encode any information.
(0.13 MB DOC)

Figure S3 Tissue-specific expression of PLN. (source: co-expressed gene database COXPRESdb: http://coxpresdb.hgc.jp. Calculation is based on the 123 human microarray experiments released by NCBI GEO version 7.)
(0.04 MB DOC)

Table S1 All SNPs with p<10–6 for the additive model from the meta-analysis of the TwinsUK, BRIGHT and DCCT/EDIC cohorts.
(0.05 MB XLS)

Table S2 Most significant SNPs from the meta-analysis of TwinsUK, BRIGHT and DCCT/EDIC cohorts within an area 20 kb upstream and downstream of the 11 known candidate genes for LQTS and SQTS.
(0.04 MB DOC)

Appendix S1 Consortium members and affiliations
(0.06 MB DOC)
