# Supplementary material for: Common Genetic Variation Near the Phospholamban Gene Is Associated with Cardiac Repolarisation: Meta-Analysis of Three Genome-Wide Association Studies
Source: PLoS One. 2009 Jul 9;4(7):e6138. doi: 10.1371/journal.pone.0006138 (PMC2704957; doi:10.1371/journal.pone.0006138)

**Figure S1: PLN protein-protein interaction network.**

This protein-protein interaction network was generated by the STRING program (http://string.embl.de) after querying the *PLN* gene using a high confidence score (0.700). Data supporting the interactions illustrated were derived from experimental studies (purple lines), databases (blue lines) and text mining (green lines). The genes that are involved in the calcium signaling pathway are indicated in red, the nodes with purple stars indicate the genes that are associated with cardiovascular diseases as based on functional annotation by DAVID (http://david.abcc.ncifcrf.gov). Only for large nodes are 3D protein structures available in STRING. The colour of the nodes does not encode any information.


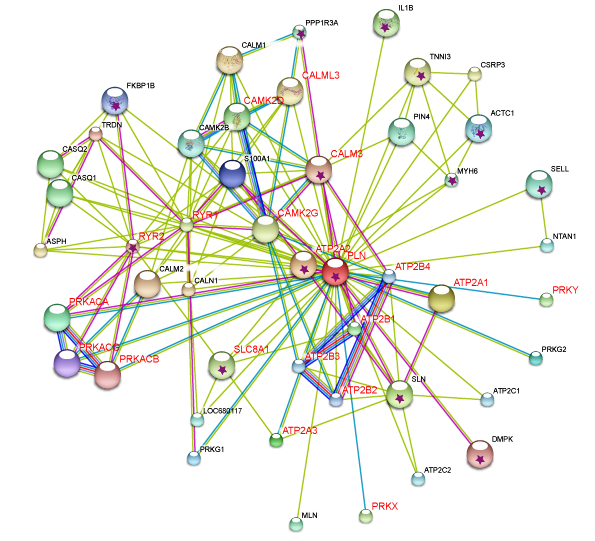

Supplement: Figure S1 — PLN protein-protein interaction network. This protein-protein interaction network was generated by the STRING program (http://string.embl.de) after querying the PLN gene using a high confidence score (0.700). Data supporting the interactions illustrated were derived from experimental studies (purple lines), databases (blue lines) and text mining (green lines). The genes that are involved in the calcium signaling pathway are indicated in red, the nodes with purple stars indicate the genes that are associated with cardiovascular diseases as based on functional annotation by DAVID (http://david.abcc.ncifcrf.gov). Only for large nodes are 3D protein structures available in STRING. The colour of the nodes does not encode any information. (0.22 MB DOC) [file pone.0006138.s001.doc]
