# Supplementary material for: Common Genetic Variation Near the Phospholamban Gene Is Associated with Cardiac Repolarisation: Meta-Analysis of Three Genome-Wide Association Studies
Source: PLoS One. 2009 Jul 9;4(7):e6138. doi: 10.1371/journal.pone.0006138 (PMC2704957; doi:10.1371/journal.pone.0006138)

**Figure S2: PLN co-expression network.**

This network is retrieved from the gene co-expression database COXPRESdb (http://coxpresdb.hgc.jp) using the PLN Entrez ID (5350) as the query. The co-expression network is drawn based on rank of correlation from 123 Human microarray experiments released by the NCBI GEO database. The bold grey lines indicate average ranks from 1 to 4. The normal light gray lines indicate average ranks from 5 to 29. The orange lines indicate conserved co-expression based on evidence from the NCBI HomoloGene database and COXPRESdb. The gene names in red indicate muscle or heart specific expression and nodes with purple stars refer to genes that are associated with cardiovascular diseases as based on functional annotation by DAVID (http://david.abcc.ncifcrf.gov). For large nodes 3D protein structures are available in STRING. The colour of the nodes does not encode any information.


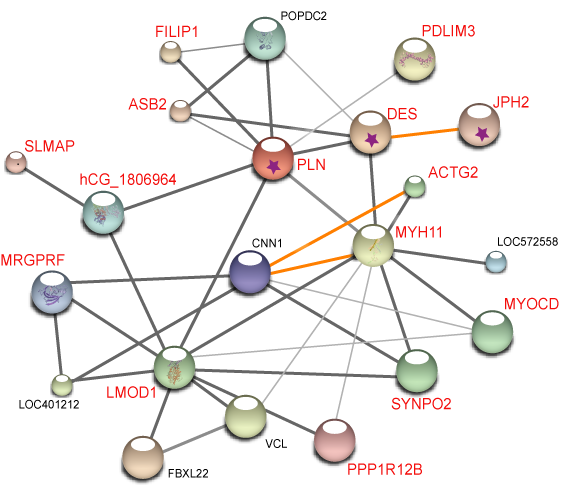

Supplement: Figure S2 — PLN co-expression network. This network is retrieved from the gene co-expression database COXPRESdb (http://coxpresdb.hgc.jp) using the PLN Entrez ID (5350) as the query. The co-expression network is drawn based on rank of correlation from 123 Human microarray experiments released by the NCBI GEO database. The bold grey lines indicate average ranks from 1 to 4. The normal light gray lines indicate average ranks from 5 to 29. The orange lines indicate conserved co-expression based on evidence from the NCBI HomoloGene database and COXPRESdb. The gene names in red indicate muscle or heart specific expression and nodes with purple stars refer to genes that are associated with cardiovascular diseases as based on functional annotation by DAVID (http://david.abcc.ncifcrf.gov). For large nodes 3D protein structures are available in STRING. The colour of the nodes does not encode any information. (0.13 MB DOC) [file pone.0006138.s002.doc]
