# Supplementary material for: Common Genetic Variation Near the Phospholamban Gene Is Associated with Cardiac Repolarisation: Meta-Analysis of Three Genome-Wide Association Studies
Source: PLoS One. 2009 Jul 9;4(7):e6138. doi: 10.1371/journal.pone.0006138 (PMC2704957; doi:10.1371/journal.pone.0006138)

**Figure S3: Tissue-specific expression of PLN.** (source: co-expressed gene database COXPRESdb: http://coxpresdb.hgc.jp. Calculation is based on the 123 human microarray experiments released by NCBI GEO version 7.)


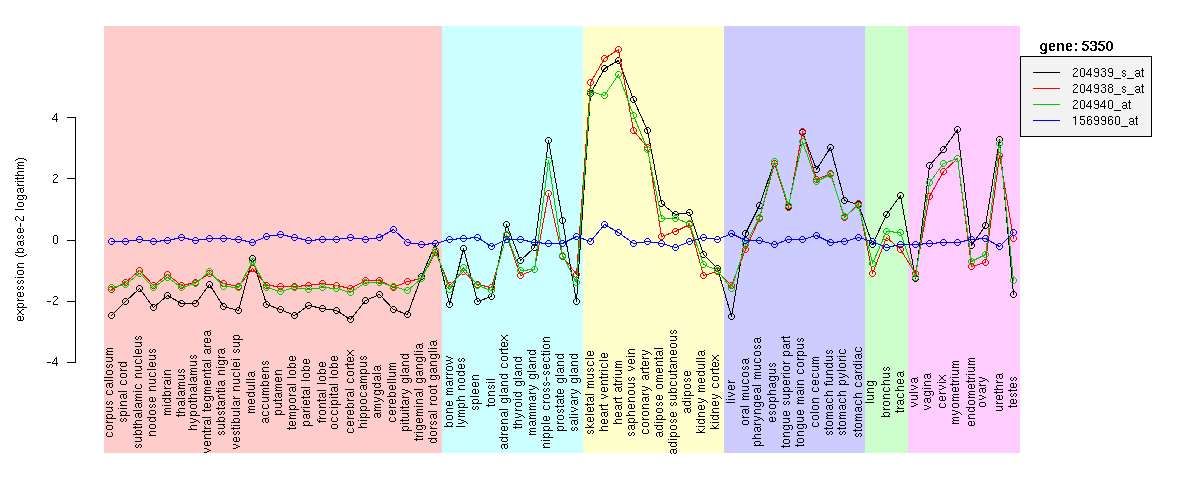

Supplement: Figure S3 — Tissue-specific expression of PLN. (source: co-expressed gene database COXPRESdb: http://coxpresdb.hgc.jp. Calculation is based on the 123 human microarray experiments released by NCBI GEO version 7.) (0.04 MB DOC) [file pone.0006138.s003.doc]
