# Supplementary material for: Common Genetic Variation Near the Phospholamban Gene Is Associated with Cardiac Repolarisation: Meta-Analysis of Three Genome-Wide Association Studies
Source: PLoS One. 2009 Jul 9;4(7):e6138. doi: 10.1371/journal.pone.0006138 (PMC2704957; doi:10.1371/journal.pone.0006138)
Supplement: Table S2 — Most significant SNPs from the meta-analysis of TwinsUK, BRIGHT and DCCT/EDIC cohorts within an area 20 kb upstream and downstream of the 11 known candidate genes for LQTS and SQTS. (0.04 MB DOC) [file pone.0006138.s005.doc]

**Table S2: Most significant SNPs from the meta-analysis of TwinsUK, BRIGHT and DCCT/EDIC cohorts within an area 20kb upstream and downstream of the 11 known candidate genes for LQTS and SQTS.**

| Gene | Chr | #SNPs | # SNPs P<0.05 | Most sig. SNP | Location^a^ | P-value (corr)^b^ |
| --- | --- | --- | --- | --- | --- | --- |
| KCNQ1 (LQT1) | 11 | 338 | 42 | rs9666604 | 2654171 | 0.0018 (0.45) |
| KCNH2 (LQT2) | 7 | 35 | 1 | rs11763131 | 150299115 | 0.0077 (0.24) |
| SCN5A (LQT3) | 3 | 112 | 2 | rs7427874 | 38618283 | 0.043 (0.99) |
| ANK2 (LQT4) | 4 | 466 | 15 | rs2279892 | 114210589 | 0.0080 (0.98) |
| KCNE1 (LQT5) | 21 | 103 | 21 | rs3787720 | 34825923 | **0.00045 (0.045)** |
| KCNE2 (LQT6) | 21 | 45 | 4 | rs2834471 | 34654347 | 0.0053 (0.21) |
| KCNJ2 (LQT7) | 17 | 42 | 3 | rs4328485 | 65689360 | 0.0091 (0.32) |
| CACNA1C (LQT8) | 12 | 667 | 3 | rs2238091 | 2592525 | 0.027 (1.00) |
| CAV3 (LQT9) | 3 | 89 | 0 | rs2268485 | 8749285 | 0.054 (0.99) |
| SCN4B (LQT10) | 11 | 69 | 7 | rs1814964 | 117512167 | 0.022 (0.79) |
| AKAP9 (LQT11) | 7 | 83 | 1 | rs17674478 | 91495889 | 0.029 (0.91) |

Chr: chromosome; sig.: significant

^a^ NCBI Genome build 36.3

^b^ P-value corrected for number of SNPs in the genic region by Šidák’s formula [65]
