# Supplementary material for: Common Genetic Variation Near the Phospholamban Gene Is Associated with Cardiac Repolarisation: Meta-Analysis of Three Genome-Wide Association Studies
Source: PLoS One. 2009 Jul 9;4(7):e6138. doi: 10.1371/journal.pone.0006138 (PMC2704957; doi:10.1371/journal.pone.0006138)
Supplement: Appendix S1 — Consortium members and affiliations (0.06 MB DOC) [file pone.0006138.s006.doc]

**Appendix:**

**WTCCC Membership and affiliations**

**Membership of the Wellcome Trust Case Control Consortium (WTCCC)**

**Management Committee:** Paul R Burton^1^, David G Clayton^2^, Lon R Cardon^3^, Nick Craddock^4^, Panos Deloukas^5^, Audrey Duncanson^6^, Dominic P Kwiatkowski^3,5^, Mark I McCarthy^3,7^, Willem H Ouwehand^8,9^, Nilesh J Samani^10^, John A Todd^2^, Peter Donnelly (Chair)^11^

**Analysis Committee:** Jeffrey C Barrett^3^, Paul R Burton^1^, Dan Davison^11^, Peter Donnelly^11^, Doug Easton^12^, David Evans^3^, Hin-Tak Leung^2^, Jonathan L Marchini^11^, Andrew P Morris^3^, I CA Spencer^11^, Martin D Tobin^1^, Lon R Cardon (Co-chair)^3^, David G Clayton (Co-chair)^2^

**UK Blood Services & University of Cambridge Controls**: Antony P Attwood^5,8^, James P Boorman^8,9^, Barbara Cant^8^, Ursula Everson^13^, Judith M Hussey^14^, Jennifer D Jolley^8^, Alexandra S Knight^8^, Kerstin Koch^8^, Elizabeth Meech^15^, Sarah Nutland^2^, Christopher V Prowse^16^, Helen E Stevens^2^, Niall C Taylor^8^, Graham R Walters^17^, Neil M Walker^2^, Nicholas A Watkins^8,9^, Thilo Winzer^8^, John A Todd^2^, Willem H Ouwehand^8,9^

**1958 Birth Cohort Controls:** Richard W Jones^18^, Wendy L McArdle^18^, Susan M Ring^18^, David P Strachan^19^, Marcus Pembrey^18,20^

**Bipolar Disorder (Aberdeen):** Gerome Breen^21^, David St Clair^21^; (**Birmingham):** Sian Caesar^22^, Katherine Gordon-Smith^22,23^, Lisa Jones^22^; **(Cardiff):** Christine Fraser^23^, Elaine K Green^23^, Detelina Grozeva^23^, Marian L Hamshere^23^, Peter A Holmans^23^, Ian R Jones^23^, George Kirov^23^, Valentina Moskvina^23^, Ivan Nikolov^23^, Michael C O’Donovan^23^, Michael J Owen^23^, Nick Craddock^23^; **(London):** David A Collier^24^, Amanda Elkin^24^, Anne Farmer^24^, Richard Williamson^24^, Peter McGuffin^24^; **(Newcastle):** Allan H Young^25^, I Nicol Ferrier^25^

**Coronary Artery Disease (Leeds):** Stephen G Ball^26^, Anthony J Balmforth^26^, Jennifer H Barrett^26^, D Timothy Bishop^26^, Mark M Iles^26^, Azhar Maqbool^26^, Nadira Yuldasheva^26^, Alistair S Hall^26^; **(Leicester):** Peter S Braund^10^, Paul R Burton^1^, Richard J Dixon^10^, Massimo Mangino^10^, Suzanne Stevens^10^, Martin D Tobin^1^, John R Thompson^1^, Nilesh J Samani^10^

**Crohn’s Disease (Cambridge):** Francesca Bredin^27^, Mark Tremelling^27^, Miles Parkes^27^; **(Edinburgh):** Hazel Drummond^28^, Charles W Lees^28^, Elaine R Nimmo^28^, Jack Satsangi^28^; **(London):** Sheila A Fisher^29^, Alastair Forbes^30^, Cathryn M Lewis^29^, Clive M Onnie^29^, Natalie J Prescott^29^, Jeremy Sanderson^31^, Christopher G Mathew^29^; **(Newcastle):** Jamie Barbour^32^, M Khalid Mohiuddin^32^, Catherine E Todhunter^32^, John C Mansfield^32^; **(Oxford):** Tariq Ahmad^33^, Fraser R Cummings^33^, Derek P Jewell^33^

**Hypertension (Aberdeen):** John Webster^34^; **(Cambridge):** Morris J Brown^35^, David G Clayton^2^; **(Evry, France): G** Mark Lathrop^36^; **(Glasgow):** John Connell^37^, Anna Dominiczak^37^; **(Leicester):** Nilesh J Samani^10^; **(London):** Carolina A Braga Marcano^38^, Beverley Burke^38^, Richard Dobson^38^, Johannie Gungadoo^38^, Kate L Lee^38^, Patricia B Munroe^38^, Stephen J Newhouse^38^, Abiodun Onipinla^38^, I Wallace^38^, Mingzhan Xue^38^, Mark Caulfield^38^; **(Oxford):** Martin Farrall^39^

**Rheumatoid Arthritis:** Anne Barton^40^, The Biologics in RA Genetics and Genomics Study Syndicate (BRAGGS) Steering Committee*, Ian N Bruce^40^, Hannah Donovan^40^, Steve Eyre^40^, Paul D Gilbert^40^, Samantha L Hider^40^, Anne M Hinks^40^, Sally L John^40^, Catherine Potter^40^, Alan J Silman^40^, Deborah PM Symmons^40^, Wendy Thomson^40^, Jane Worthington^40^

**Type 1 Diabetes:** David G Clayton^2^, David B Dunger^2,41^, Sarah Nutland^2^, Helen E Stevens^2^, Neil M Walker^2^, Barry Widmer^2,41^, John A Todd^2^

**Type 2 Diabetes (Exeter):** Timothy M Frayling^42,43^, Rachel M Freathy^42,43^, Hana Lango^42,43^, John R B Perry^42,43^, Beverley M Shields^43^, Michael N Weedon^42,43^, Andrew T Hattersley^42,43^; **(London):** Graham A Hitman^44^; **(Newcastle):** Mark Walker^45^; **(Oxford):** Kate S Elliott^3,7^, Christopher J Groves^7^, Cecilia M Lindgren^3,7^, Nigel W Rayner^3,7^, Nicholas J Timpson^3,46^, Eleftheria Zeggini^3,7^, Mark I McCarthy^3,7^

**Tuberculosis (Gambia):** Melanie Newport^47^, Giorgio Sirugo^47^; **(Oxford):** Emily Lyons^3^, Fredrik Vannberg^3^, Adrian VS Hill^3^

**Ankylosing Spondylitis:** Linda A Bradbury^48^, Claire Farrar^49^, Jennifer J Pointon^48^, Paul Wordsworth^49^, Matthew A Brown^48,49^

**AutoImmune Thyroid Disease:** Jayne A Franklyn^50^, Joanne M Heward^50^, Matthew J Simmonds^50^, Stephen CL Gough^50^

**Breast Cancer:** Sheila Seal^51^, Breast Cancer Susceptibility Collaboration (UK)*, Michael R Stratton^51,52^, Nazneen Rahman^51^

**Multiple Sclerosis:** Maria Ban^53^, An Goris^53^, Stephen J Sawcer^53^, Alastair Compston^53^

**Gambian Controls (Gambia):** David Conway^47^, Muminatou Jallow^47^, Melanie Newport^47^, Giorgio Sirugo^47^; **(Oxford):** Kirk A Rockett^3^, Dominic P Kwiatkowski^3,5^

**DNA, Genotyping, Data QC and Informatics (Wellcome Trust Sanger Institute, Hinxton):** Claire Bryan^5^, Suzannah J Bumpstead^5^, Amy Chaney^5^, Kate Downes^2,5^, Jilur Ghori^5^, Rhian Gwilliam^5^, Sarah E Hunt^5^, Michael Inouye^5^, Andrew Keniry^5^, Emma King^5^, Ralph McGinnis^5^, Simon Potter^5^, Rathi Ravindrarajah^5^, Pamela Whittaker^5^, David Withers^5^, Panos Deloukas^5^; **(Cambridge):** Hin-Tak Leung^2^, Sarah Nutland^2^, Helen E Stevens^2^, Neil M Walker^2^, John A Todd^2^

**Statistics (Cambridge):** Doug Easton^12^, David G Clayton^2^; **(Leicester):** Paul R Burton^1^, Martin D Tobin^1^; **(Oxford):** Jeffrey C Barrett^3^, David Evans^3^, Andrew P Morris^3^, Lon R Cardon^3^; **(Oxford):** Niall J Cardin^11^, Dan Davison^11^, Teresa Ferreira^11^, Joanne Pereira-Gale^11^, Ingeleif B Hallgrimsdóttir^11^, Bryan N Howie^11^, Jonathan L Marchini^11^, I CA Spencer^11^, Zhan Su^11^, Yik Ying Teo^3,11^, Damjan Vukcevic^11^, Peter Donnelly^11^

**PIs:** David Bentley^5,54^, Matthew A Brown^48,49^, Lon R Cardon^3^, Mark Caulfield^38^, David G Clayton^2^, Alistair Compston^53^, Nick Craddock^23^, Panos Deloukas^5^, Peter Donnelly^11^, Martin Farrall^39^, Stephen CL Gough^50^, Alistair S Hall^26^, Andrew T Hattersley^42,43^, Adrian VS Hill^3^, Dominic P Kwiatkowski^3,5^, Christopher G Mathew^29^, Mark I McCarthy^3,7^, Willem H Ouwehand^8,9^, Miles Parkes^27^, Marcus Pembrey^18,20^, Nazneen Rahman^51^, Nilesh J Samani^10^, Michael R Stratton^51,52^, John A Todd^2^, Jane Worthington^40^

*^1^ Genetic Epidemiology Group, Department of Health Sciences, University of Leicester, Adrian Building, University Road, Leicester, LE1 7RH, UK; ^2^ Juvenile Diabetes Research Foundation/Wellcome Trust Diabetes and Inflammation Laboratory, Department of Medical Genetics, Cambridge Institute for Medical Research, University of Cambridge, Wellcome Trust/MRC Building, Cambridge, CB2 0XY, UK; ^3^ Wellcome Trust Centre for Human Genetics, University of Oxford, Roosevelt Drive, Oxford OX3 7BN, UK; ^4^ Department of Psychological Medicine, Henry Wellcome Building, School of Medicine, Cardiff University, Heath Park, Cardiff CF14 4XN, UK; ^5^ The Wellcome Trust Sanger Institute, Wellcome Trust Genome Campus, Hinxton, Cambridge CB10 1SA, UK; ^6^ The Wellcome Trust, Gibbs Building, 215 Euston Road, London NW1 2BE, UK; ^7^ Oxford Centre for Diabetes, Endocrinology and Medicine, University of Oxford, Churchill Hospital, Oxford, OX3 7LJ, UK; ^8^ Department of Haematology, University of Cambridge, Long Road, Cambridge, CB2 2PT, UK; ^9^ National Health Service Blood and Transplant, Cambridge Centre, Long Road, Cambridge, CB2 2PT, UK; ^10^ Department of Cardiovascular Sciences, University of Leicester, Glenfield Hospital, Groby Road, Leicester, LE3 9QP, UK; ^11^ Department of Statistics, University of Oxford, 1 South Parks Road, Oxford OX1 3TG, UK; ^12^ Cancer Research UK Genetic Epidemiology Unit, Strangeways Research Laboratory, Worts Causeway, Cambridge CB1 8RN, UK^; 13^ National Health Service Blood and Transplant, Sheffield Centre, Longley Lane, Sheffield S5 7JN, UK; ^14^ National Health Service Blood and Transplant, Brentwood Centre, Crescent Drive, Brentwood, CM15 8DP, UK; ^15^ The Welsh Blood Service, Ely Valley Road, Talbot Green, Pontyclun, CF72 9WB, UK; ^16^ The Scottish National Blood Transfusion Service, Ellen’s Glen Road, Edinburgh, EH17 7QT, UK; ^17^ National Health Service Blood and Transplant, Southampton Centre, Coxford Road, Southampton, SO16 5AF, UK; ^18^ Avon Longitudinal Study of Parents and Children, University of Bristol, 24 Tyndall Avenue, Bristol, BS8 1TQ, UK; ^19^ Division of Community Health Services, St George’s University of London, Cranmer Terrace, London SW17 0RE, UK; ^20^ Institute of Child Health, University College London, 30 Guilford St, London WC1N 1EH, UK; ^21^ University of Aberdeen, Institute of Medical Sciences, Foresterhill, Aberdeen, AB25 2ZD, UK; ^22^ Department of Psychiatry, Division of Neuroscience, Birmingham University, Birmingham, B15 2QZ, UK; ^23^ Department of Psychological Medicine, Henry Wellcome Building, School of Medicine, Cardiff University, Heath Park, Cardiff CF14 4XN, UK; ^24^ SGDP, The Institute of Psychiatry, King’s College London, De Crespigny Park Denmark Hill London SE5 8AF, UK; ^25^ School of Neurology, Neurobiology and Psychiatry, Royal Victoria Infirmary, Queen Victoria Road, Newcastle upon Tyne, NE1 4LP, UK; ^26^ LIGHT and LIMM Research Institutes, Faculty of Medicine and Health, University of Leeds, Leeds, LS1 3EX, UK; ^27^ IBD Research Group, Addenbrooke’s Hospital, University of Cambridge, Cambridge, CB2 2QQ, UK; ^28^ Gastrointestinal Unit, School of Molecular and Clinical Medicine, University of Edinburgh, Western General Hospital, Edinburgh EH4 2XU UK; ^29^ Department of Medical & Molecular Genetics, King’s College London School of Medicine, 8^th^ Floor Guy’s Tower, Guy’s Hospital, London, SE1 9RT, UK; ^30^ Institute for Digestive Diseases, University College London Hospitals Trust, London, NW1 2BU, UK; ^31^ Department of Gastroenterology, Guy’s and St Thomas’ NHS Foundation Trust, London, SE1 7EH, UK; ^32^ Department of Gastroenterology & Hepatology, University of Newcastle upon Tyne, Royal Victoria Infirmary, Newcastle upon Tyne, NE1 4LP, UK; ^33^ Gastroenterology Unit, Radcliffe Infirmary, University of Oxford, Oxford, OX2 6HE, UK^; 34^ Medicine and Therapeutics, Aberdeen Royal Infirmary, Foresterhill, Aberdeen, Grampian AB9 2ZB, UK; ^35^ Clinical Pharmacology Unit and the Diabetes and Inflammation Laboratory, University of Cambridge, Addenbrookes Hospital, Hills Road, Cambridge CB2 2QQ, UK; ^36^ Centre National de Genotypage, 2, Rue Gaston Cremieux, Evry, Paris 91057.; ^37^ BHF Glasgow Cardiovascular Research Centre, University of Glasgow, 126 University Place, Glasgow, G12 8TA, UK; ^38^ Clinical Pharmacology and Barts and The London Genome Centre, William Harvey Research Institute, Barts and The London, Queen Mary’s School of Medicine, Charterhouse Square, London EC1M 6BQ, UK; ^39^ Cardiovascular Medicine, University of Oxford, Wellcome Trust Centre for Human Genetics, Roosevelt Drive, Oxford OX3 7BN, UK; ^40^arc Epidemiology Research Unit, University of Manchester, Stopford Building, Oxford Rd, Manchester, M13 9PT, UK; ^41^ Department of Paediatrics, University of Cambridge, Addenbrooke’s Hospital, Cambridge, CB2 2QQ, UK; ^42^ Genetics of Complex Traits, Institute of Biomedical and Clinical Science, Peninsula Medical School, Magdalen Road, Exeter EX1 2LU UK; ^43^ Diabetes Genetics, Institute of Biomedical and Clinical Science, Peninsula Medical School, Barrack Road, Exeter EX2 5DU UK; ^44^ Centre for Diabetes and Metabolic Medicine, Barts and The London, Royal London Hospital, Whitechapel, London, E1 1BB UK; ^45^ Diabetes Research Group, School of Clinical Medical Sciences, Newcastle University, Framlington Place, Newcastle upon Tyne NE2 4HH, UK; ^46^ The MRC Centre for Causal Analyses in Translational Epidemiology, Bristol University, Canynge Hall, Whiteladies Rd, Bristol BS2 8PR, UK; ^47^ MRC Laboratories, Fajara, The Gambia; ^48^ Diamantina Institute for Cancer, Immunology and Metabolic Medicine, Princess Alexandra Hospital, University of Queensland, Woolloongabba, Qld 4102, Australia; ^49^ Botnar Research Centre, University of Oxford, Headington, Oxford OX3 7BN, UK; ^50^ Department of Medicine, Division of Medical Sciences, Institute of Biomedical Research, University of Birmingham, Edgbaston, Birmingham B15 2TT, UK; ^51^ Section of Cancer Genetics, Institute of Cancer Research, 15 Cotswold Road, Sutton, SM2 5NG, UK; ^52^ Cancer Genome Project, The Wellcome Trust Sanger Institute, Wellcome Trust Genome Campus, Hinxton, Cambridge CB10 1SA, UK; ^53^ Department of Clinical Neurosciences, University of Cambridge, Addenbrooke’s Hospital, Hills Road, Cambridge CB2 2QQ, UK; ^54^ PRESENT ADDRESS: Illumina Cambridge, Chesterford Research Park, Little Chesterford, Nr Saffron Walden, Essex, CB10 1XL, UK.*

**QTSCD Membership:**

Arne Pfeufer ^1,2^, Serena Sanna^3^, Dan E Arking^4^, Martina Mu¨ller ^5–7^, Vesela Gateva^8^,

Christian Fuchsberger^9^, Georg B Ehret^4^, Marco Orru´ ^3^, Cristian Pattaro^9^, Anna Ko¨ttgen^10^, Siegfried Perz^11^, Gianluca Usala^3^, Maja Barbalic^12^, Man Li^10^, Benno Pu¨tz^13^, Angelo Scuteri^14^, Ronald J Prineas^15^, Moritz F Sinner^7^, Christian Gieger^5^, Samer S Najjar^16^, W H Linda Kao^10^, Thomas W. Mu¨hleisen^17,18^, Mariano Dei^3^, Christine Happle^1,2^, Stefan Mo¨hlenkamp^19^, Laura Crisponi^3^, Raimund Erbe^l19^,

Karl-Heinz Jo¨ckel^20^, Silvia Naitza^3^, Gerhard Steinbeck^7^, Fabio Marroni^9^, Andrew A Hicks^9^, Edward Lakatta^16^, Bertram Mu¨ller-Myhsok^13^, Peter P Pramstaller^9,21,22^, H-Erich Wichmann^5,6^, David Schlessinger^23^, Eric Boerwinkle^12^, Thomas Meitinger^1,2^, Manuela Uda^3^, Josef Coresh^10,24^, Stefan Ka¨a¨b^7^, Goncalo R Abecasis^8^ & Aravinda Chakravarti^4,24^

^1^ Institute of Human Genetics, Technical University Munich, Germany.

^2^Institute of Human Genetics, Helmholtz Center Munich, Germany.

^3^Istituto di Neurogenetica e Neurofarmacologia, CNR, Monserrato, Cagliari, Italy. ^4^McKusick-Nathans Institute of Genetic Medicine, Johns Hopkins University, Baltimore, Maryland, USA.

^5^Institute of Epidemiology, Helmholtz Center Munich, Germany.

^6^Institute of Informatics, Biometry and Epidemiology, Ludwig-Maximilians-Universitat, Munich, Germany.

^7^Department of Medicine I, Klinikum Grosshadern, Munich, Germany.

^8^Center for Statistical Genetics, Department of Biostatistics, University of Michigan, Ann Arbor, Michigan, USA.

^9^Institute of Genetic Medicine, EURAC European Academy, Bolzano, Italy. ^10^Department of Epidemiology, Johns Hopkins University, Baltimore, Maryland, USA. ^11^Institute of Medical Informatics, Helmholtz Center Munich, Germany.

^12^Genetics Center, University of Texas Health Science Center, Houston, Texas, USA. ^13^Statistical Genetics, Max Planck Institute of Psychiatry, Munich, Germany.

^14^Unita` Operativa Geriatria, Istituto Ricovero e Cura per Anziani, Rome, Italy.

^15^Wake Forest University School of Medicine, Winston-Salem, North Carolina, USA. ^16^Laboratory of Cardiovascular Science, Gerontology Research Center, National Institute on Aging, Baltimore, Maryland, USA.

^17^Department of Genomics, Life & Brain Center, University of Bonn, Bonn, Germany. ^18^Institute of Human Genetics, University of Bonn, Bonn, Germany.

^19^Clinic of Cardiology, West German Heart Center, University Hospital of Essen, University Duisburg-Essen, Germany.

^20^Institute for Medical Informatics, Biometry and Epidemiology, University Hospital of Essen, University Duisburg-Essen, Germany.

^21^Department of Neurology, General Central Hospital, Via Bohler 5, Bolzano, Italy. ^22^Department of Neurology, University of Lu¨ beck, Lu¨ beck, Germany.

^23^Laboratory of Genetics, National Institute on Aging, Baltimore, Maryland, USA. ^24^Department of Medicine, Johns Hopkins University, Baltimore, Maryland, USA.

**QTGEN Membership:**

Christopher Newton-Cheh^1–3^, Mark Eijgelsheim^4^, Kenneth M Rice^5^, Paul I Wde Bakker^2,6^, Xiaoyan Yin^3,7^, Karol Estrada^8^, Joshua C Bis^9,10^, Kristin Marciante^9,10^, Fernando Rivadeneira^4,8^, Peter A Noseworthy^1^, Nona Sotoodehnia^9,11^, Nicholas L Smith^9,12,13^, Jerome I Rotter^14^, Jan A Kors^15^, Jacqueline CM Witteman^4,16^, Albert Hofman^4,16^, Susan R Heckbert^9,12,17^, Christopher J O’Donnell^3,18,19^, Andre´ G Uitterlinden^4,8,16^, Bruce M Psaty^10,12,17,20^, Thomas Lumley^5^, Martin G Larson^3,7^, & Bruno H Ch Stricker^4,8,16,20^

^1^Center for Human Genetic Research, Cardiovascular Research Center, Massachusetts General Hospital, Boston, Massachusetts, USA.

^2P^rogram in Medical and Population Genetics, Broad Institute of Harvard and MIT, Cambridge, Massachusetts, USA.

^3^National Heart, Lung and Blood Institute’s Framingham Heart Study, Framingham, Massachusetts, USA.

^4^Department of Epidemiology, Erasmus Medical Center, Rotterdam, The Netherlands. ^5^Department of Biostatistics, University of Washington, Seattle, Washington, USA. ^6^Division of Genetics, Department of Medicine, Brigham and Women’s Hospital, Boston, Massachusetts, USA.

^7^Department of Mathematics and Statistics, Boston University, Boston, Massachusetts, USA.

^8^Department of Internal Medicine, Erasmus Medical Center, Rotterdam, The Netherlands.

^9^Cardiovascular Health Research Unit, University of Washington, Metropolitan Park East Tower, Seattle, Washington, USA.

^10^Department of Medicine, University of Washington, Seattle, Washington, USA. ^11^Division of Cardiology, Department of Medicine, University of Washington School of Medicine, Seattle, Washington, USA.

^12^Department of Epidemiology, University of Washington, Seattle, Washington, USA. ^13^Seattle Epidemiologic Research Center, Veterans Administration Office of Research and Development, Seattle, Washington, USA.

^14^Medical Genetics Institute, Cedars-Sinai Medical Center, Los Angeles, California, USA.

^15^Department of Medical Informatics, Erasmus Medical Center, Rotterdam, The Netherlands.

^16^Netherlands Genomics Initiative-sponsored Netherlands Consortium for Healthy Aging,

PO Box 2040, 3000 CA Rotterdam, The Netherlands.

^17^Center for Health Studies, Group Health, Seattle, Washington, USA.

^18^National Heart, Lung and Blood Institute, Bethesda, Maryland, USA.

^19^Cardiology Division, Massachusetts General Hospital, Boston, Massachusetts, USA. ^20^Department of Health Services, University of Washington, Seattle, Washington, USA.
